# Quality of Life in Spanish advanced non-small-cell lung cancer patients: determinants of global QL and survival analyses

**DOI:** 10.1186/s40064-016-2559-9

**Published:** 2016-06-22

**Authors:** Juan Ignacio Arraras, Berta Hernandez, Maite Martinez, Koldo Cambra, Mikel Rico, Jose Juan Illarramendi, Antonio Viudez, Berta Ibañez, Uxue Zarandona, Enrique Martinez, Ruth Vera

**Affiliations:** Medical Oncology Department, Complejo Hospitalario de Navarra, Irunlarrea 3, 31008 Pamplona, Spain; Radiotherapeutic Oncology Department, Complejo Hospitalario de Navarra, Irunlarrea 3, 31008 Pamplona, Spain; Departamento de Ciencias, Universidad Pública de Navarra - UPNA, Campus de Arrosadía, 31006 Pamplona, Navarra Spain; Red de Investigación en Servicios Sanitarios en Enfermedades Crónicas (REDISSEC), Fundación Miguel Servet-NavarraBiomed, Irunlarrea 3, 31008 Pamplona, Spain

**Keywords:** Lung cancer, Advanced, Quality of Life, Survival, Determinants

## Abstract

**Purpose:**

This paper studies the Quality of Life (QL) of Spanish advanced non-small-cell lung cancer (NSCLC) patients receiving platinum-doublet chemotherapy, compares our results with those from studies from other cultural areas, and identifies factors associated with global QL and survival prognostic variables.

**Methods:**

EORTC QLQ-C30 and QLQ-LC13 questionnaires were completed three times by 39 patients along treatment and follow-up. Univariate and multivariate logistic regression analyses were performed to study global QL determinants (≤50 points considered low global-QL score). Analyses of prognostic variables for death were performed (Cox proportional hazards models).

**Results:**

QL mean scores in the whole sample were moderately high, with limitations (>30) in physical, role, social functioning, emotional areas, fatigue, pain, neuropathy and global QL. Differences with studies from other cultural areas were mainly found in the lower score for dyspnoea (≥15 points). There were no significant differences in QL scores between the first and second assessments. In six areas, the third assessment was lower than the first and second: fatigue, hair loss (>20 points); physical, social functioning, neuropathy (10–20 points); emotional functioning (5–10 points). The best model to explain the chances of low QL includes, as explanatory variables, high emotional functioning as protective factor and fatigue as risk factor (R^2^ = 0.70). Eight QL areas (four pain-related) and performance status showed a statistically significant association with survival.

**Conclusion:**

Patients adapted well to their disease and treatments. Platinum-doublet can be administered in advanced NSCLC patients. Our QL data are in line with those from other cultural areas.

## Background

Lung cancer is the leading cause of cancer-associated mortality worldwide (Li et al. 2012). Non-small-cell lung cancer (NSCLC) is more frequently diagnosed in advanced stages, when patients present symptoms and treatment side-effects that may affect their Quality of Life (QL; Arrieta et al. 2013). Larsson et al. (2012) found that before the start of treatment almost all QL areas (both those common to others tumours and those specifically for lung cancer) were statistically deteriorated in advanced NSCLC patients in comparison with the general population.

QL assessment and improvement have a key role in oncology. Since the treatment of advanced NSCLC can have a limited effect on survival, maintaining or improving the QL of advanced NSCLC patients are considered important treatment goals (Wintner et al. 2013).

Braun et al. (2011) consider that treatment for advanced lung cancer must always be judged in the context of its effects on patient QL. In this respect, chemotherapy is understood that might increase survival and QL in the advanced-disease non-small-cell lung cancer (Grønberg et al. 2010). Platinum-based chemotherapy and taxanes may have a positive effect on survival and QL in advanced NSCLC patients with a good performance status (Bosch-Barrera et al. 2011; Von Plessen et al. 2006).

Larsson et al. (2012) consider that the effect of the disease and treatment on the QL of cancer patients depends on several factors (including the patient’s performance status, age, and level of fatigue) and state there is a need for more research on the determinants of the QL of advanced NSCLC patients. The role of QL as a prognostic survival factor in cancer (including lung cancer) has been studied in recent years (Grande et al. 2009). Several discrepancies have been found (Braun et al. 2011; Quinten et al. 2014) that could be due to factors like the wide variety of QL questionnaires administered or including patients with different tumour sites in the same study. Arrieta et al. (2013) believe that the role of depression as a prognostic factor in NSCLC is debatable. Further studies on QL as a prognostic factor in NSCLC could prove useful. Clinical and biographical variables have also been studied as prognostic factors (Grønberg et al. 2010). Studies of QL determinants and survival prediction factors in advanced disease may bring important benefits, such as improvements in patient management and the provision of better information (Grande et al. 2009).

Cross-cultural differences play a key role in important areas for cancer patients, such as information (Mystakidou et al. 2004) or QL: Kaptein et al. (2011), for example, found cross-cultural differences in the QL scores of NSCLC patients.

In this paper we study QL and its changes during treatment and follow-up in advanced-disease NSCLC Spanish patients treated with platinum-doublet, compare our results with those from studies conducted in other cultural areas, and analyse the determinants of the patients’ global QL as well as factors related to survival.

## Methods

### Participants

A consecutive sample of lung cancer patients who initiated treatment at the Medical Oncology Department of the Complejo Hospitalario de Navarra (Spain) between August 2009 and June 2012 were invited to participate in the study.

Inclusion criteria were stage IV or stage III-B NSCLC [patients ineligible for curative radiotherapy according to the previous AJCC staging system (Mountain 1997)] and start of chemotherapy. The patients were treated with platinum-doublet (cisplatin or carboplatin depending on level of comorbidity and performance status) combined with taxanes, gemcitabine or pemetrexed. Not other systemic treatment was administered. Cycles were repeated every 3 weeks. Four to six cycles of platinum-doublet were proposed for each patient. Patients with non-squamous histology had subsequent maintenance treatment with pemetrexed. Exclusion criteria were a cognitive state that did not permit treatment evaluation, a life expectancy of <3 months, a performance status <60 [Karnosky scale (Karnofsky and Burchenal 1949)] or positive EGFR or ALK when diagnosed.

### Measures

All patients completed the EORTC questionnaires QLQ-C30 version 3.0 (Aaronson et al. 1993) and QLQ-LC13 (Bergman et al. 1994), which our group had validated for use in Spain (Arraras et al. 2000, 2002). The structures of these questionnaires are shown in Table 1. QLQ-C30 evaluates areas common to various tumour sites and treatments, whereas QLQ-LC13 evaluates areas associated with lung cancer and its treatments. Questionnaires with under 70 % of the items answered were excluded.

**Table 1:** Sociodemographic and clinical characteristics of the sample

Characteristics	No	Percentage	Mean	SD
Present age (range 40–81)			66.1	9.9
≥66 years old	24	61.5		
Gender				
Female	4	10.3		
Male	35	89.7		
Marital status				
Single	1	2.5		
Married	36	92.3		
Widowed	2	5.2		
Limiting comorbidity	19	48.7		
Performance status (range 60–90)^a^			79.1	8.9
60–80	26	66.7		
90	13	33.3		
Stage				
IV	35	89.7		
III-B	4	10.3		
Histological types				
Adenocarcinoma	18	46.2		
Large-cell carcinoma	1	2.5		
Squamous cell carcinoma	19	48.8		
Adenosquamous carcinoma	1	2.5		
Chemotherapy				
Carboplatin doublet	29	74.4		
Cisplatin doublet	10	25.6		
Morphine	29	74.4		
2nd assessment				
Performance status (range 30–100)			70.5	17.4
Treatment response				
Progressive disease	17	43.6		
Stable disease	14	35.9		
Partial response	8	20.5		
Toxicity				
Neutropenia	2	5.2		
Thrombopenia	2	5.2		
Vomiting	1	2.6		
Fatigue	1	2.6		
3rd assessment				
Performance status (range 50–90)			76.8	12.1
Treatment response 3rd assessment				
Progression	7	36.8		
No changes	12	63.2		
Chemotherapy cycles received (range 1–14) median 4			5.2	
Platin doublet	33	15.4		
Platin doublet + maintenance treatment	6	84.6		
CT from 3 to 2 weeks	3	7.7		
Time-to-disease progression				
Range 0.93–17.23; median 5.20			5.98	4.54
Survival				
Range 2.20–44.63; median 7.45			10.99	8.92

^a^The median of the Performance status in the first measurement is presented as scores did not follow a normal distribution

Treatment response 2nd, 3rd assessments: treatment response at second and third assessment points

Toxicity: ≥3 values

Chemotherapy cycles received by those who completed the third assessments

CT from 3 to 2 weeks: patients whose CT changed from being administered every 3 weeks to every 2 weeks

Time to progression and survival in months

The treating physician assessed toxicity levels at the second and third assessments through selected items from the Common Terminology Criteria for Adverse Events (CTCAE) Version 4.0 (National Cancer Institute 2009), performance status using the Karnofsky scale (Karnofsky and Burchenal 1949; in the three assessments), and comorbidity (in the initial assessment).

### Data collection procedures

Patients were invited to complete the QL questionnaires at three points during the treatment and follow-up periods: on the first day of chemotherapy, 2 weeks after the third cycle, and at the follow-up consultation 6 weeks after the end of the platinum-doublet treatment. Patients did not know the status of their disease (stable or progressing) when completing the second and third assessments. Questionnaires were administered in paper pencil version. Patients filled in the instruments at a desk in the Oncology Departments. A psychologist was present while the questionnaires were being completed. After completing their questionnaires, the patients were able to discuss their answers with the psychologist. Patients did not need help to understand and to reply to the questions.

### Ethics and consent

This study was conducted in accordance with the recommendations of the Declaration of Helsinki and approved by the Ethics Committee of the Complejo Hospitalario de Navarra (Number 107/2009). Informed consent was obtained from all individual participants included in the study.

### Statistical analysis

Descriptive statistics were used to summarize the sample characteristics and the QL scores. Changes between the three measurements in QL scores were studied using the Friedman test. The Wilcoxon test with the Bonferroni criteria was used to determine between which pairs of measurements the differences appeared. Differences in QL mean scores were calculated between the pairs of assessments that had shown statistically significant differences after Bonferroni correction (these QL mean score differences were considered ‘less-than-a-little’ if the change was <5 points, ‘little’ if it was 5–10 points, ‘moderate’ if it was 10–20, and ‘very much’ if it was >20 points; Osoba et al. 1998). For functional areas changes towards higher scores imply a QL improvement; for symptom areas, changes to higher scores indicate a QL decrease.

To identify which patients’ characteristics were related to low global QL, univariate logistic regression analyses were performed before the start of treatment with the categorized scores as response variables and sociodemographic (age, gender and civil status), clinical (performance status and limiting comorbidity) and the other QL areas as explanatory variables. Here we considered a score ≤50 as low global QL. To complement these analyses, we also used multivariate logistic regression models using the backward regression method and including those areas found to be statistically significant in univariate logistic regression.

Analyses of prognostic variables were also conducted. First, the relationship between the clinical and demographic factors (age, gender, civil status, limiting comorbidity, chemotherapy protocol—cisplatin or carboplatin—and performance status) and baseline QL, with overall survival (OS), was analysed (univariate Cox proportional hazards models). Then the QL statistically significant baseline scores and performance status were adjusted for gender and age. Hazard ratios were calculated for 10-point differences in QL and performance status scores (clinically meaningful important difference; Osoba et al. 1998). OS was measured from the date of the first assessment to the date of death due to cancer. Patients who died due to another cause or were still alive at the time of analyses were censored. A *p* value ≤0.05 was regarded as statistically significant in all the analyses performed. The SPSS 21 program was used in these analyses.

## Results

46 patients were eligible. Three of them declined to participate in the study. A total of 39 patients (out of 43 initial candidates who gave informed consent) completed the first assessment. Reasons for not completing this assessment were death (two patients) and derivation to the palliative care unit (two patients). A total of 34 patients completed the second assessment and 19 patients completed the third. The reason for not completing the second assessment was disease progression (five patients). The reasons for not completing the third assessment were switches to another protocol (three patients) and stop the chemotherapy treatment (12 patients: nine due to disease progression and three due to toxicity). The sociodemographic and clinical characteristics of the patients are shown in Table 1. Mean age was 66.1, 35 patients (89.7 %) were male, and also 35 patients were in stage IV. In the case of three patients, chemotherapy treatment (CT) passed from being administered every 3 weeks to every 2 weeks due to toxicity.

QL mean scores for the QLQ-C30 and the QLQ-LC13 are presented in Table 2. Moderate QL limitations (>30) occurred in: emotional functioning, fatigue and global QL (in all three assessments); sleep disturbance (in the first and second assessments); physical functioning, pain and neuropathy (in the second assessment); and role and social functioning (in the second and third assessments). Light QL affectations (between 20 and 29 points) appeared in: physical, role and social functioning, and pain (in the first measurement), appetite loss, constipation and coughing (in the first and second assessments); cognitive functioning and pain elsewhere (in the second assessment); dyspnoea and hair loss (in the second and third assessments); and physical functioning, sleep disturbance and neuropathy (in the third assessment).

**Table 2:** Mean scores for QLQ-C30 and QLQ-LC13 areas and association with global QL

	1st assessment	2nd assessment	3rd assessment	p
	Mean (SD) (N = 39)	Mean (SD) (N = 34)	Mean (SD) (N = 19)	
QLQ-C30 areas				
Physical^a^	74.7 (26.5)	60.6 (32.7)	70.2 (23.7)	0.004
Role^a^	70.1 (36.9)	51.9 (37.9)	62.3 (29.8)	0.055
Emotional^a^	64.1 (28.6)	57.6 (32.2)	64.9 (29.4)	0.029
Cognitive^a^	82.9 (24.0)	75.0 (28.5)	80.7 (21.7)	0.146
Social^a^	76.5 (30.9)	57.8 (36.5)	64.9 (28.8)	0.004
Global^a^	60.0 (25.0)	53.4 (26.8)	60.5 (21.7)	0.148
Fatigue^b^	35.0 (30.4)	57.1 (56.7)	44.4 (27.9)	0.047
Nausea^b^	6.4 (15.6)	7.5 (17.9)	7.0 (18.7)	0.779
Pain^b^	27.4 (31.6)	33.8 (36.8)	17.5 (24.5)	0.187
Dyspnoea^b^	12.8 (22.4)	12.7 (18.4)	12.3 (19.9)	0.016
Sleep disturbance^b^	34.2 (38.6)	37.2 (36.5)	22.8 (24.9)	0.933
Appetite loss^b^	26.5 (32.6)	29.4 (36.5)	12.3 (27.8)	0.741
Constipation^b^	24.8 (32.5)	28.4 (35.9)	14.1 (23.1)	0.149
Diarrhoea^b^	9.5 (21.5)	3.9 (15.9)	3.1 (15.3)	0.819
Financial impact^b^	8.5 (25)	13.7 (28.6)	0 (0)	0.607
QLQ-LC13 areas				
Coughing^b^	28.2 (30.1)	22.5 (26.9)	8.8 (15.1)	0.148
Dyspnoea^b^	13.7 (19.9)	22.2 (23.8)	21.1 (22.5)	0.063
Haemoptysis^b^	2.6 (8.9)	2.9 (8.6)	1.7 (7.7)	0.607
Sore mouth^b^	8.5 (25.1)	18.6 (29.2)	14.0 (20.2)	0.021
Dysphagia^b^	5.9 (15.1)	15.7 (28.7)	10.5 (19.4)	0.091
Peripheral neuropathy^b^	10.3 (17.4)	31.4 (14.7)	29.8 (21.9)	0.002
Hair loss^b^	5.1 (19.5)	21.6 (27.1)	26.3 (21.0)	0.001
Chest pain^b^	10.3 (18.9)	18.6 (24.9)	12.3 (16.5)	0.641
Pain in arm or shoulder^b^	12.8 (22.4)	14.7 (24.9)	10.5 (22.4)	0.179
Other pain sites^b^	18.8 (28.4)	20.2 (32.2)	3.5 (10.5)	0.247

p: Level of significance Friedman test, based on complete cases only

^a^The scores range from 0 to 100, where a higher score represents a higher functional level

^b^The scores range from 0 to 100, where a higher score represents a greater degree of symptoms

Eight areas (physical, emotional and social functioning, fatigue, dyspnoea, sore mouth, peripheral neuropathy and hair loss) showed differences when the three assessments were compared (Table 2) but these dropped to six after Bonferroni correction. In all of these six areas there was a worse QL in the third assessment compared to the other two (for functional areas it implies lower scores; for symptom areas, higher scores). There were no significant differences between the first and second assessments. Differences between the first and the third assessment appeared in physical and social functioning and neuropathy (moderate change) and fatigue and hair loss (very much change). Differences between the second and the third assessment appeared in physical functioning and fatigue (moderate change) and emotional functioning (little change).

A statistically significant relationship was found between the chances of low global QL and performance status (p = 0.033; OR 0.91, 95 % CI 0.83–0.99, R^2^ = 0.19). No other statistically significant relationship was found between global QL and any of the clinical or demographic variables.

The QL areas with a statistically significant relationship with chances of low global QL and the highest R2 were role, emotional and cognitive functioning (higher values were associated with higher global QL), and fatigue, dyspnoea and appetite loss(higher values were associated with lower global QL; see Table 3). The multivariate model that best explains the chances of low global QL is a combination of emotional functioning as a protective effect (p = 0.006; OR 0.93, 95 % CI 0.88–0.98) and fatigue as a risk factor (p = 0.015; OR 1.06, 95 % CI 1.01–1.12; R^2^ = 70).

**Table 3:** QLQ-C30 and QLQ-LC13 areas with a significant relationship with the chances of low global QL

	OR (95 % CI)	R^2^	p value
QLQ-C30 areas			
Role^a^	0.96 (0.94–0.99)	0.32	0.006
Emotional^a^	0.93 (0.89–0.97)	0.53	0.001
Cognitive^a^	0.95 (0.91–0.99)	0.28	0.015
Fatigue^b^	1.06 (1.02–1.10)	0.46	0.002
Pain^b^	1.03 (1.00–1.06)	0.18	0.039
Dyspnoea^b^	1.06 (1.01–1.12)	0.28	0.016
Appetite loss^b^	1.04 (1.01–10.07)	0.28	0.011
QLQ-LC13 areas			
Coughing^b^	1.03 (1.00–1.06)	0.19	0.030
Dyspnoea^b^	1.08 (1.02–1.15)	0.33	0.016

^a^The scores range from 0 to 100, where a higher score represents a higher functional level

^b^The scores range from 0 to 100, where a higher score represents a greater degree of symptoms

The results of the univariate Cox regression analyses indicate that performance status and ten QL areas were significantly associated with survival. Eight of these areas and performance status showed a statistically significant association with survival after adjustment for gender and age [physical functioning (p = 0.004), role functioning (p = 0.022), fatigue (p = 0.003), pain (p = 0.002), appetite loss (p = 0.029), sore mouth (p = 0.008), pain arm or shoulder (p = 0.024), other pain sites (p = 0.005; Table 4)]. Four of these QL areas evaluate pain. Higher scores (in 10-point units) in performance status and functional areas (physical and role functioning) were associated with a lower risk of death, and higher scores in symptoms areas (fatigue, pain, appetite loss, sore mouth, pain arm or shoulder, and other pain sites) were associated with a higher risk of death.

**Table 4:** Univariate Cox regression analyses of survival at base line

Explanatory variables	Unadjusted^a^		Adjusted^b^	
	HR (95 % CI)	p	HR (95 % CI)	p
Socio-demographic and clinical variables				
Age (continuous)	0.98 (0.95–1.01)	0.282		
Gender (male vs. female)	1.92 (0.66–5.56)	0.229		
Civil status (married vs. other status)	0.97 (0.29–3.20)	0.966		
Limiting comorbidity (yes vs. no)	1.13 (0.71–2.40)	0.386		
Chemotherapy protocol (carboplatin vs. cisplatin)	0.65 (0.32–1.13)	0.230		
Performance status (continuous)	0.95 (0.92–0.99)	0.018	0.59 (0.39–0.88)	0.010
QLQ-C30 areas				
Physical^c^	0.83 (0.72–0.96)	0.011	0.81 (0.71–0.93)	0.004
Role^c^	0.86 (0.77–0.96)	0.006	0.87 (0.77–0.99)	0.022
Emotional^c^	0.96 (0.87–1.11)	0.804	1.01 (0.88–1.17)	0.853
Cognitive^c^	0.85 (0.73–0.99)	0.044	0.88 (0.73–1.06)	0.169
Social^c^	0.91 (0.82–1.01)	0.099	0.93 (0.82–1.06)	0.268
Global^c^	0.90 (0.76–1.06)	0.210	0.88 (0.73–1.07)	0.203
Fatigue^d^	1.25 (1.09–1.43)	0.002	1.25 (1.08–1.44)	0.003
Nausea^d^	1.08 (0.97–1.34)	0.490	0.93 (0.67–1.31)	0.697
Pain^d^	1.26 (1.20–1.44)	0.001	1.26 (1.09–1.45)	0.002
Dyspnoea^d^	1.03 (0.90–1.18)	0.620	1.04 (0.90–1.19)	0.602
Sleep disturbance^d^	0.99 (0.92–1.08)	0.939	1.01 (0.92–1.11)	0.818
Appetite loss^d^	1.14 (1.03–1.26)	0.008	1.13 (1.10–1.27)	0.029
Constipation^d^	1.03 (0.95–1.13)	0.422	1.03 (0.94–1.14)	0.495
Diarrhoea^d^	1.01 (0.86–1.18)	0.930	1.04 (0.89–1.22)	0.607
Financial impact^d^	1.19 (1.03–1.37)	0.019	1.16 (0.99–1.34)	0.600
QLQ-LC13 areas				
Coughing^d^	1.01 (0.91–1.14)	0.755	1.02 (0.91–1.15)	0.709
Dyspnoea^d^	1.17 (0.97–1.42)	0.104	1.14 (0.94–1.39)	0.191
Haemoptysis^d^	1.37 (0.94–1.98)	0.099	1.39 (0.95–2.03)	0.090
Sore mouth^d^	1.25 (1.05–1.49)	0.010	1.27 (1.06–1.52)	0.008
Dysphagia^d^	1.16 (0.94–1.43)	0.167	1.19 (0.97–1.47)	0.099
Peripheral neuropathy^d^	1.04 (0.86–1.27)	0.655	1.04 (0.85–1.27)	0.702
Hair loss^d^	1.08 (0.92–1.25)	0.355	1.07 (0.91–1.25)	0.434
Chest pain^d^	1.07 (0.89–1.29)	0.425	1.02 (0.85–1.23)	0.815
Pain in arm or shoulder^d^	1.19 (1.01–1.40)	0.034	1.22 (1.03–1.45)	0.024
Other pain sites^d^	1.18 (1.03–1.33)	0.012	1.20 (1.06–1.36)	0.005

*HRs* hazard ratios, *CI* confidence interval

^a^Unadjusted: Univariate Cox proportional hazards models

^b^Adjusted: Univariate Cox proportional hazards models adjusted for gender and age

^c^The scores range from 0 to 100, where a higher score represents a higher functional level

^d^The scores range from 0 to 100, where a higher score represents a greater degree of symptoms

## Discussion

The main results of this study are that QL scores in a sample of advanced disease NSCLC Spanish patients treated with platinum-doublet were moderately high. In six QL areas there was a worse QL in the third assessment compared to the other two (for functional areas it implies lower scores; for symptom areas, higher scores) and there were no significant differences between the latter two assessments. A logistic model with emotional functioning and fatigue has been created to explain the chances of global QL. Cox regression analyses indicated that performance status and eight QL areas were statistically significantly associated with survival after adjustment for gender and age.

The high proportion of patients (93 %) who agreed to participate in the study and the fact that the reasons for not completing the questionnaires were all medical suggest that the study was feasible and well accepted by patients receiving treatment, and this can be regarded as strength of the study. Among the limitations, we should mention the fact that all of the patients come from the same hospital, the low statistical power due to: the small number of participants; the proportion of patients for whose 2nd and 3rd assessments have not been possible; and also the high number of tests done due to the amount of QLQ areas to be analized, which has been only partially corrected in the pairwise comparisons with the Bonferroni correction. We might consider the results of the present study as preliminary. It would be interesting to repeat this study in other centres to try to confirm its results.

The demographic and clinical characteristics of the sample were representative of patients treated at the Complejo Hospitalario de Navarra.

QL scores were satisfactory if we take into account the advanced disease stages. The QL limitations in the first assessment suggest that the patients in the study were in a good condition to receive the treatment.

The first day of treatment scores were in line with those of other studies conducted with the EORTC questionnaires in NSCLC patients from other cultural areas in which QL had been administered just before starting treatment: Scandinavian countries (Larsson et al. 2012—334 patients; Braun et al. 2011—1194 patients; Grønberg et al. 2010—402 patients), the Netherlands (24 patients) and Japan (22 patients, Kaptein et al. 2011) and Korea [Park et al. 2013—139 patients; in most of these studies the patients were at advanced disease stages (Larsson et al. 2012; Braun et al. 2011; Grønberg et al. 2010; Kaptein et al. 2011)]. It is important when making these comparisons to take into account the limitation of the low sample size in our study. These scores were also in line with those from the EORTC reference values manual for NSCLC at various stages (Scott et al. 2008). Patients in our study showed less dyspnoea (≥15 points) than those in the six above studies and the EORTC reference values. This could be due to the morphine-based treatment. Limitations in fatigue were moderate but lower (≥10 points) than in NSCLC Scandinavian patients at the same (Larsson et al. 2012; Grønberg et al. 2010) disease stages.

No significant changes were found between the first day of treatment and during-treatment assessments. Few changes appeared between the third and the first two assessments, mainly in treatment-related areas. These results are in line with those of other studies conducted with the EORTC questionnaires. Wintner et al. (2013) found no significant QL changes during the CT period in Austrian advanced lung-cancer patients. Von Plessen et al. (2006) found a worsening in the third assessment compared to the first in fatigue (moderate change) and global QL (little change) in Scandinavian advanced NSCLC patients. Park et al. (2013) also found few changes in the third assessment compared to the first in early-stage NSCLC Korean patients who had received adjuvant CT (neuropathy and hair loss; very much change).

The QL scores in each assessment, the low number of changes in these QL scores, the low number of patients who did not complete the second assessment, the low number of patients who reduced CT dose (from 3-week cycles to 2-week cycles), and the low toxicity scores suggest that toxicity control was satisfactory.

Performance status was found to be a determinant of global QL in other studies of advanced NSCLC patients (Larsson et al. 2012) and lung cancer patients(NSCLC and small cell lung cancer) at various disease stages (Mohan et al. 2007).

Fatigue and emotional functioning were found to be key determinants of global QL in other studies conducted with the EORTC questionnaires. Ostlund et al. (2007) found that a combination of emotional functioning and fatigue was the main global QL determinant in lung cancer patients (at a variety of disease stages). Arrieta et al. (2013) also found that emotional functioning was associated with global QL in advanced NSCLC patients. Emotional functioning (Cramarossa et al. 2013) and fatigue (Beijer et al. 2008) were found to be important determinants of global QL in advanced disease cancer patients with different tumor sites.

Ostlund et al. (2007) suggest that low emotional functioning can influence global QL scores because it includes negative thoughts than can be reference points for patients when assessing their QL. Fatigue is considered to have a major impact on the QL of cancer patients because it includes physical, emotional and cognitive dimensions (Weis et al. 2013) that can affect every aspect of the patient’s daily life (Ostlund et al. 2007).

Performance status was found to be a predictor of survival in other studies with NSCLC patients at the same disease stages as those in our study (Ediebah et al. 2014)^,^ and at a variety of stages (Li et al. 2012).

Age was not a predictive factor in our study, in other studies of NSCLC patients at the same stages of disease (Arrieta et al. 2013; Ediebah et al. 2014) or in a review of studies of lung cancer patients (Quinten et al. 2014). Braun et al. (2011), on the other hand, found age to be a predictive factor in NSCLC patients at a variety of stages (though their age range was wider).

Comorbidity was not found to be a predictive factor in other studies of patients with advanced NSCLC (Grønberg et al. 2010; Bergman et al. 1994; Maione et al. 2005). Grønberg et al. (2010) consider comorbidity to be a predictive factor of survival in NSCLC at early stages but that this has not been confirmed at advanced stages when patients may die of cancer before they die from their other illnesses. Comorbidity has been assessed in other studies performed in NSCLC patients with the Simplified Comorbidity Score (SCS; Colinet et al. 2005). This instrument has shown to predict survival in NSCLC patients in different disease stages, and might be used in future studies with just advanced NSCLC patients to assess its role as predictive factor.

Our results on QL as predictive factors are in line with those of other studies in which the EORTC questionnaires were administered. Physical, role functioning, fatigue and pain (in our case also in the pain areas of the QLQ-LC13) were also found to be predictive factors in advanced NSCLC patients (Grande et al. 2009; Ediebah et al. 2014) and at a variety of stage (Li et al. 2012; Braun et al. 2011). Limitations in these four areas could be a consequence of disease progression.

Appetite loss was found to be a predictor of survival in advanced NSCLC patient (Grande et al. 2009) at a variety of stages (Li et al. 2012; Braun et al. 2011). Appetite loss may be a predictive factor as it could be part of the anorexia-cachexia syndrome that frequently appears in advanced lung cancer patients (Del Ferraro et al. 2012).

Cognitive functioning was not found to be a predictor of survival. This could be because patients with cognitive limitations were excluded from the study.

Global QL was not found to be a predictive factor in our study or in that of Grande et al. (2009) with advanced NSCLC patients. However, it was in other NSCLC studies at a variety of stages (Li et al. 2012; Braun et al. 2011). A larger sample could provide more solid conclusions.

Nausea and vomiting, diarrhea, neuropathy and hair loss were not found to be predictors. These variables may be related to treatment side effects. Emotional functioning, sleep disturbance, constipation, financial impact and chest pain were not found to be predictors in studies conducted with advanced NSCLC patients (Grande et al. 2009; Ediebah et al. 2014).

Scores for dyspnoea, haemoptysis and dysphagia were low in our sample, which could explain why they were not predictive factors. Coughing was not a predictive factor in studies with NSCLC patients at various stages (Li et al. 2012).

## Conclusions

Advanced Spanish NSCLC patients treated with platinum-doublet showed moderate QL scores during treatment and follow-up assessments. When studying the patients who have filled in the three assessments, their QL scores were worse in the third assessment, which indicates that the patients adapted well to their disease and treatment. QL and clinical data suggest that platinum-doublet can be administered in advanced NSCLC patients. Our QL data are in line with those from other cultural areas.

Longitudinal QL assessments are important in advanced lung cancer patients because the data they provide could, for example, help to assess more patient areas and enable early recognition of arising symptom aggravation, thus leading to more efficient and more timely interventions (Wintner et al. 2013).

In this study the QL factors related to global QL and survival we have identified are in line with those found in other studies. Further research could help to assess what role the management of QL determinants and QL survival prognostic factors could play in the treatment of cancer patients (Arrieta et al. 2013).

## Abbreviations

QL: Quality of Life
NSCLC: non-small-cell lung cancer
CT: chemotherapy treatment
CTCAE: Common Terminology Criteria for Adverse Events
